# Using Support Vector Machine Ensembles for Target Audience Classification on Twitter

**DOI:** 10.1371/journal.pone.0122855

**Published:** 2015-04-13

**Authors:** Siaw Ling Lo, Raymond Chiong, David Cornforth

**Affiliations:** School of Design, Communication and Information Technology, The University of Newcastle, Callaghan, NSW 2308, Australia; University of Warwick, UNITED KINGDOM

## Abstract

The vast amount and diversity of the content shared on social media can pose a challenge for any business wanting to use it to identify potential customers. In this paper, our aim is to investigate the use of both unsupervised and supervised learning methods for target audience classification on Twitter with minimal annotation efforts. Topic domains were automatically discovered from contents shared by followers of an account owner using Twitter Latent Dirichlet Allocation (LDA). A Support Vector Machine (SVM) ensemble was then trained using contents from different account owners of the various topic domains identified by Twitter LDA. Experimental results show that the methods presented are able to successfully identify a target audience with high accuracy. In addition, we show that using a statistical inference approach such as bootstrapping in over-sampling, instead of using random sampling, to construct training datasets can achieve a better classifier in an SVM ensemble. We conclude that such an ensemble system can take advantage of data diversity, which enables real-world applications for differentiating prospective customers from the general audience, leading to business advantage in the crowded social media space.

## Introduction

In the age of social media, companies can no longer rely on advertisements or press releases to reach out to their customers. Instead, it is essential for companies to actively listen to and engage with their online audience in order to reveal business transparency and human touch. A recent study [1] found that nearly 80% of consumers would more likely be interested in a company because of its brand’s presence on social media. It is therefore not surprising that 77% of the Fortune 500 companies have active Twitter accounts and 70% of them maintain active Facebook accounts to engage with their potential customers [2].

Even though the 1.28 billion active user base [3] of Twitter, Facebook and other social media platforms can be a valuable source of information for any business, it is not an easy feat to identify a target audience in the crowded social media space. This is mainly because of the challenge of extracting commercially viable contents from the vast amount of free-form conversations. Hence, automated machine learning approaches that can help in classifying and identifying the target audience on social media platforms will be highly beneficial to business owners.

Due to the privacy policy of Facebook profiles, our work focuses on Twitter, where most of the contents and activities shared online are open and available. Twitter allows its registered users or account owners to send and read short messages (up to 140 characters) called *tweets*. Twitter users may subscribe to or follow other users’ tweets and thus, the subscribers are also known as *followers*. However, it is not uncommon for Twitter account owners to have people subscribing to their accounts through a marketing campaign or free product samples offered. These people may not be genuine followers, and may not be truly interested in contents shared by account owners they subscribed to. It is hence of interest for a Twitter account owner who is a business owner to distinguish (i.e., to classify) among those who are genuine and those who are not, thereby being able to identify a target audience from its list of followers so that appropriate offers can be effectively sent to the right audience. The subject or business company selected for this study is *Samsung Singapore* or “samsungsg” (its Twitter account name).

Supervised learning, or classification, is a type of machine learning that attempts to determine some relationship between a set of input vectors that represent stimuli, and a corresponding set of values on a nominal scale that represents a category or a class. The relationship is obtained by applying an algorithm to training samples that are 2-tuples *<u*, *z>*, consisting of an input vector *u* and a class label *z*. The learned relationship can then be applied to instances of *u* not included in the training set, in order to discover the corresponding class label *z* [4]. For social media data, the training set requires annotation in order to identify the class before training can commence.

It is well known that constructing an annotated training dataset is one of the biggest challenges in using a supervised machine learning approach. Due to the vast amount and diverse nature of Twitter followers’ tweets, it is not feasible to manually annotate the tweets for training a machine learning model. We reason that tweets from an account owner can be used to build a positive training dataset, as the group of followers who are tweeting similar contents (within a similar period of time) is more likely to comprise the target audience compared to others who are not sharing similar contents. This would save us from the need to manually annotate the vast amount of tweets from the followers and is more practical if the approach is to be adopted in a real-world application.

While it may be logical to use the account owner’s tweets as the positive training dataset, it can be challenging to construct a negative training dataset of similar nature without the need of annotation. The automated identification of groups within a dataset is known as unsupervised learning, because unlike supervised learning, annotations or class labels are not available. As our study focuses on classifying the target audience from the list of followers, it is of interest to analyse if an unsupervised topic modelling approach, such as Latent Dirichlet Allocation (LDA) [5], can be used to discover topics or domains of interest of the followers and construct negative training datasets based on the domains uncovered. In other words, the negative training dataset can be built using tweets from other account owners who are actively sharing contents in the topics or domains (other than the target domain) shared by the followers. However, this approach would result in a dataset imbalance situation, as the total number of representative tweets of different domains can be large compared to the account owner’s tweets, due to the diverse nature of the domains shared by the followers. Assuming that there are 10 domains, we have a situation of 1:10 imbalance in the training dataset, where there is only one part of the positive training dataset (which consists of the tweets shared by the target account owner) but 10 parts of the negative training dataset (which are the tweets from other domains).

Although the data imbalance issue and diversity of various domains may pose a challenge to any classifiers, it can be an advantage when multiple classifier algorithms are trained and then combined (i.e., an ensemble system). The main reason is due to the fact that the success of an ensemble system depends heavily on the diversity of the classifiers and hence the idea of using the contents from account owners of different domains should provide classifiers with different outputs and improve the overall classification performance. A strategic combination of these classifiers can reduce the total error with the intuition that the ensemble system will learn from each of the classifiers as they make different kinds of errors. Specifically, an ensemble system needs classifiers whose decision boundaries are adequately different from each other to achieve a better classification result.

The diversity can be achieved via a number of methods [6]. The most popular method is to use different training datasets to train individual classifiers. Another approach is to use different training parameters for different classifiers or use entirely different types of classifiers. Besides that, diversity can also be attained using different features or different subsets of existing features. Although there are many ways on how ensemble systems can be constructed, we focus on using different training datasets to train individual classifiers, to assess if tweet contents of different account owners can be used to identify the target audience from the list of followers.

Due to the different sizes of the imbalance training dataset, we can either under-sample the majority class or over-sample the minority class, so as to balance it. The simplest way is to under-sample either through random selection or pre-defined sampling based on certain criteria. This method risks information loss in the majority class and hence may not fully capture all features of the class. Besides that, predicting with such a machine learning algorithm may lead to low accuracies [7]. The other method is over-sampling the minority class either through duplication of the original dataset or sampling with replacement of the minority data. The caveat is that although over-sampling does not cause any information loss, it can introduce an unnatural bias in favour of the minority class. Various approaches such as random sampling and bootstrapping are therefore implemented to assess if there is any effect in choosing different approaches for constructing the training dataset.

Since we would like to ascertain if the content of an account owner (i.e., samsungsg) can be used to classify and identify the target audience from its list of followers, resampling methods such as random sampling, bootstrapping and ensemble methods such as majority vote, bagging and stacking are adopted, where balancing the dataset is the emphasis instead of modifying the classifiers. As a result, all the classifiers are of the same type and use the same parameters. The Support Vector Machine (SVM) [8] is chosen as the main classifier for evaluation purposes due to its known ability in text classification [9] and best performance in benchmark text categorisation collection [10].

The main contributions of this work can be summarised as follows:

- We introduce an approach that leverages on both unsupervised (Twitter LDA) and supervised (SVM ensembles) learning methods on tweets for target audience classification.
- To the best of our knowledge, our work in this paper is the first attempt to classify a target audience from the list of followers of an account owner on Twitter using both Twitter LDA and SVM ensembles with minimum manual annotation efforts required.
- From our observation of the results, it is essential to adopt some statistical inference approach such as bootstrapping in over-sampling instead of using random sampling to construct training datasets to achieve a better classifier in an SVM ensemble. The ensemble method using bagging achieves the best performance compared to other methods.
- Owners’ tweet contents can potentially be used as the training dataset in machine learning for target audience identification.

In the next section, we describe our approach using Twitter LDA and SVM ensembles. Following which, we outline the experimental setup and evaluate the results. After that, we discuss our findings along with some related work in target audience classification and SVM ensembles before concluding the paper.

## A New Approach for Target Audience Classification

In this section, we present the approach of combining unsupervised (Twitter LDA) and supervised (SVM ensembles) learning to classify a target audience from the list of followers of an account owner (i.e., samsungsg) with minimum annotation efforts. First, we introduce Twitter LDA [11] and describe how the domains of the followers are discovered based on their tweets using this unsupervised learning method. Then, we explain how the various SVM ensembles are constructed to take advantage of data diversity from the various domains uncovered.

### Discovery of Followers’ Domains using Twitter LDA

**Fig 1:**
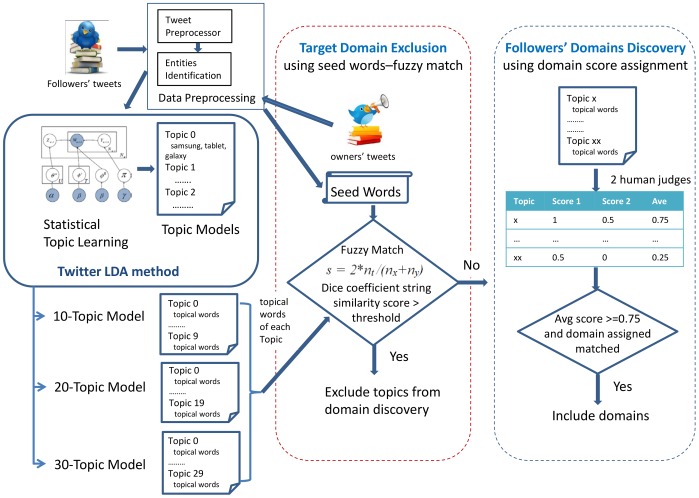
Followers’ domains discovery using Twitter LDA.

LDA, a renowned generative probabilistic model for topic discovery, has recently been used in various social media studies [11][12]. LDA uses an iterative process to build and refine a probabilistic model of documents, each containing a mixture of topics. However, standard LDA may not work well with Twitter, as tweets are typically very short. If one aggregates all the tweets of a follower to increase the size of the documents, this may diminish the fact that each tweet is usually about a single topic. As such, we have adopted the implementation of Twitter LDA [11] for unsupervised topic discovery. The procedure of how Twitter LDA is used in followers’ domains discovery is given in Fig 1.

As can be seen in the figure, followers’ tweets are first pre-processed to identify the relevant entities or phrases before they are passed to Twitter LDA for topic learning and clustering. The topical words identified by each topic model are compared to seed words generated using the account owner’s tweets. Topics with topical words matched above the threshold are excluded for further processing, as they are identified as target domains. The remaining topics and their topical words are then manually assessed by two human judges, so that a domain and a score can be assigned for each topic. Topics with consistent domains assigned and having scores above 0.75 are selected to be included in the potential followers’ domains that will be used for extracting negative training data for the SVM ensemble.

#### Twitter LDA

To uncover meaningful topics from followers’ tweets, a selection of topic numbers have to be determined. A range of topic models were tested in our preliminary study [13], and it was found that topic models from 10 to 30 (with an interval of 10) can sufficiently be used for potential target audience identification. Consequently, we have chosen three topic models (i.e., 10, 20 and 30) in this study. We ran these three different topic models for 100 iterations of Gibbs sampling while keeping the model parameters or Dirichlet priors constant: *α* = 0.5, *β*_*d*_ = 0.01, *β*_*b*_ = 0.01 and *γ* = 20.

For each of the topic models, 10 topical words have been selected to represent each topic group. For example, the topic model with 10 topics will generate 10 topic groups and each topic group contains top 10 topical words according to the distribution probability of Twitter LDA. As three different topic models have been used in this study, there are a total of 60 (10+20+30) topic groups generated. Even though the topic discovery process is unsupervised through Twitter LDA, we have used a semi-supervised method to assign topic groups to various topic categories. Two human judges have been asked to assign a score to each topic group according to the top-10 topic words, and an average of the assigned scores is allocated to each topic group. The scores consist of 1 if a domain is identified, 0.5 if there are multiple domains or noisy words, and 0 if no domain is identified.

#### Target Domain Exclusion using Seed Words-Fuzzy Match

It is understandable that the topic groups identified can contain topical words that are highly related to the content from the account owner or target domain. Therefore, topic groups with contents similar to the tweets shared by the account owner should be excluded so that other domains can be identified more effectively and subsequently used as the negative training dataset. One of the approaches to solve the target domain exclusion problem is to define a set of ontology, keywords or seed words that are relevant to the account owner. This can be done manually with the help of domain experts. However, the process is fully dependent on the availability and knowledge of a domain expert, which can be a challenge as the assessment and assignment of seed words can vary among the experts. As such, it is of interest to explore if the content shared by the account owner can be used to extract seed words automatically. With the use of the seed words, related topic groups can be identified through fuzzy matching to the topical words of topic groups, and those topic groups with matching words are eventually excluded before the analysis of followers’ domains by the human judges. Details of seed words generation, the fuzzy match method and data pre-processing procedures can be found in S1 Methods under the Supporting Information section.

#### Followers’ Domains Discovery

The 60 topic groups identified by Twitter LDA first need to be processed to exclude the topic groups that are related to the account owner through the seed words-fuzzy match approach. The remaining topic groups are then assigned to the human judges independently. Besides assigning a score to the topic group, each of the human judges also annotates the group with a relevant domain. Examples of domains are music, sports, politics, daily musing, etc. An average value is then generated for each topic group and the combination of the annotated domains is selected as the domain for the topic group. We only consider a topic group as a potential domain of the followers when its average score is 0.75 and above. The main reason is because an average score of 0.5 or less indicates that one or more of the human judges are unsure of the topic and hence it is less convincing to select the topic group as one of the representative domains.

In our study, eight domains or topics of interest have been discovered from the analysis of Twitter LDA using tweets from the list of samsungsg followers. These are daily musing, food, sports (football), Singapore related news, marketing, music, transport, and news. The average scores of the topic groups discovered are shown in Table 1 and the representative topical words are listed in the same table.

**Table 1:** The domains identified from followers’ tweets using Twitter LDA.

**Topic Model**	**Topic Group Id**	**Annotated Domain**	**Topical words**	**Average Score**
10	Topic 9	Daily musing	love, people, life, god, things, feel	1
20	Topic 6	Food	singapore, food, lunch, dinner, coffee, tea, chicken	1
	Topic 7	Football, English premier league (EPL)	united, manchester, league, chelsea, david, goal	1
	Topic 8	Daily musing	people, love, life, things, god, feel	1
	Topic 12	Singapore related	singapore, airport, points, club, changi	0.75
	Topic 0	Daily musing	happy, video, birthday, love, mothers	0.75
30	Topic 10	Daily musing	day, good, happy, morning, mothers, birthday, dinner	1
	Topic 15	Daily musing	time, work, sleep, school, long	1
	Topic 18	Daily musing	people, life, love, happy, things, god	1
	Topic 28	Football, EPL	chelsea, league, united, match, madrid	1
	Topic 1	Social media marketing	social, media, marketing, twitter, facebook, business	0.75
	Topic 14	Music	singapore concert, tour, fans, tickets, album	0.75
	Topic 16	Transport	singapore, mrt, blk, bus, wifi	0.75
	Topic 25	News	indonesia, model, tokyo, festival	0.75

Even though relevant followers’ tweets of the identified domains have the potential to be used as the training dataset, our study focuses on using tweets from account owners of these domains to assess if it is possible to build a classifier that is able to predict or identify the target audience with minimum annotation efforts. This is based on the fact that a preliminary study [14] has shown that classifiers built using followers’ tweets in training data do not perform as well as training data built using an account owner’s tweets. Besides that, one of the main benefits of using training datasets constructed from account owners is that they are mostly well defined within their domains, and this approach eliminates the need to annotate the contents of the followers, which can be of huge volume and may contain many noisy features. As quite a few identified topic groups from samsungsg have been annotated as daily musing, three account owners have been selected to represent this domain. They are joannepeh (daily musing/celebrity), kiasuparents (daily musing/parenting) and tiongbahruplaza (daily musing/shopping). The rest of the representative Twitter account owners include hungrygowhere (food), premierleague (football), tocsg (Singapore related contents and news), belindaang (marketing), mtvasia (music), sgdrivers (traffic) and SGnews (news). These 10 account owners have been selected as they are the popular Twitter accounts in Singapore according to online Twitter analytic tools such as wefollow.com.

### SVM Ensembles

Due to the diversity of the domains identified from the follower tweets, it is of interest to study if an ensemble of machine learning algorithms is able to make use of the different decision boundaries generated from the individual classifiers (using a training dataset based on the various domains) to strategically combine the classification results and hence achieve a better performance than is possible with a single classifier. In this study, we focus on the capability of SVM ensembles in classifying the target audience from the list of followers. In the ensuing section, we first introduce the SVM. This is then followed by another two sections describing the bootstrapping method and various ensemble algorithms, respectively.

#### The SVM

The SVM is a supervised learning approach for two- or multi-class classification and it has been used successfully in many applications, including text categorisation [9]. It separates a given known set of {+1, -1} labelled training data via a hyperplane that is maximally distant from the positive and negative samples. This optimally separating hyperplane in the feature space corresponds to a non-linear decision boundary in the input space. More details of the SVM can be found in [8].

Consider a set of *N* distinct samples (x_*i*_, y_*i*_) with x_*i*_∈ℜ^*D*^ and y_*i*_∈ℜ^*d*^. An SVM is modelled as

$$\sum_{i}a_{i}K\left(x,x_{i}\right)+b,i\in \left[1,N\right]$$ (1)

where *K*(x, x_*i*_) is the kernel function, and *α* and *b* are the parameter and threshold of the SVM, respectively.

The LibSVM implementation of RapidMiner [15] has been used in this study, and the sigmoid kernel type is selected as it produces higher precision prediction than other kernels such as the radial basis function or polynomial kernel. Since the training input of an SVM is through a matrix of feature vectors, the feature vectors used in this study are created using term frequency analysis of the tweet contents after data pre-processing (see S1 Methods for details) as well as word stemming using Porter [16]. Specifically, we have identified and used a total of 1239 features in creating the training feature vectors.

As the number of tweets shared by each follower is different, a *v* score is calculated by aggregating the classification results from each individual tweet of each follower’s tweet set. The final assignment of the *v* score is based on the following representation:

$$v=n_{s}/n_{t}$$ (2)

where *n*_*s*_ is the number of tweets that are classified as positive by the SVM and *n*_*t*_ is the total number of tweets shared by a follower. If 5 tweets out of a total of 50 tweets of a particular follower are classified as positive, then the *v* score assigned is 5/50 = 0.01. The total number of tweets is used to normalise the score instead of an average value of all tweets. This is done so that the resulting score is more capable of representing the true interest of the follower. For example, if follower1 tweeted 2 related tweets out of a total of 10 tweets, the *v* score assigned will be 0.2. While the *v* score for follower2 is 0.02 if only 2 related tweets are classified as positive out of a total of 100 tweets. This is in contrast to using an average value, as both follower1 and follower2 will be assigned the same *v* score that may not fully represent their interests.

#### Bootstrapping Using a Single SVM Model

Bootstrapping [17] is a common method used to address the imbalance data issue through resampling of the minority class via replacement. As our study takes into consideration the temporal effect, the amount of tweets that we can obtain is limited to the number of tweets shared by the various owners within a 6-month period. As a result, it is not possible to collect as many samples to avoid the pitfall of either risking information loss in the majority class or introducing bias in favour of the minority class.

**Fig 2:**
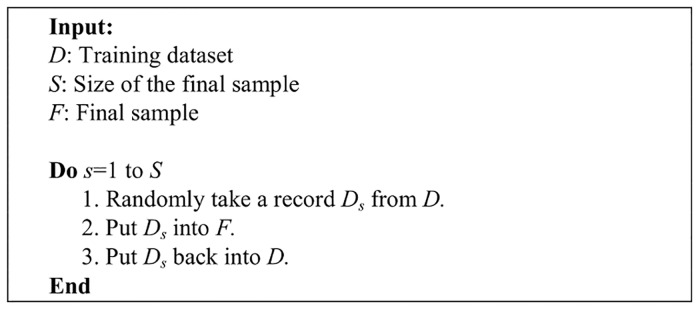
The bootstrapping algorithm.

Bootstrap sampling uses a computation approach instead of traditional distributional assumptions, and it adopts a non-parametric approach to statistical inference so that the sample distribution can be better estimated than merely duplicating the sample. A pseudocode description of bootstrapping is provided in Fig 2.

**Fig 3:**
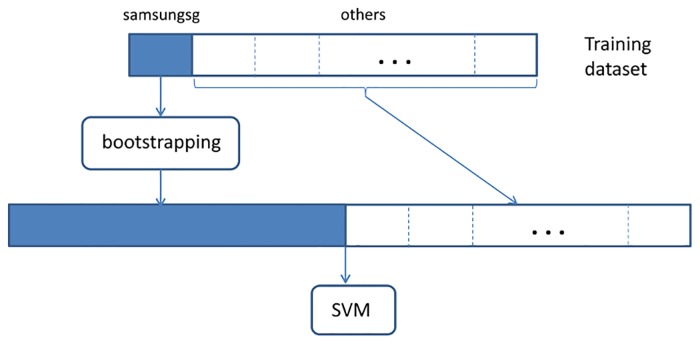
A general architecture of bootstrapping using a single SVM model.

In this study, the tweets of the account owner, samsungsg, have been resampled through bootstrapping to construct a training dataset that is of similar size as the training datasets generated using tweets of 10 account owners from the other domains. Details of the training dataset and the general architecture can be found in Table 2 and Fig 3, respectively.

**Table 2:** The configuration of bootstrapping using a single SVM model.

**Method**	**Training dataset** ^a^	**Configuration**
SVM with bootstrapping sampling	samsungsg (1978) and others (1978)	1 SVM model

^a^The number in the brackets represents the number of records. The data collection and pre-processing process can be found in S1 Methods. 1978 records from 10 different domains have been extracted as the ‘others’ training dataset. Due to the consideration of temporal effect, only the past 200 records or less from each domain in the same period (as samsungsg) have been extracted.

#### Ensembles Using Multiple SVM Models

**Fig 4:**
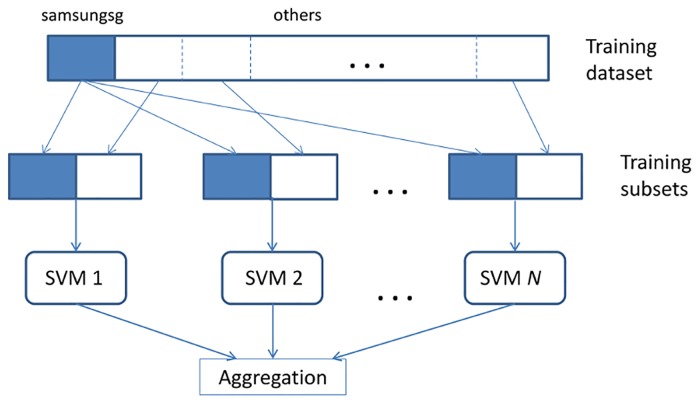
A general architecture of the ensemble system using multiple SVM models.

As one of the focuses of this study is to ascertain if the tweets from account owners can be used to identify the target audience from the list of followers, it is of interest to assess if the ensemble of classifiers built from the training datasets of the various domains can perform better than the common bootstrapping method mentioned earlier. After all, the success of an ensemble system depends largely on the diversity of the classifiers that make up the ensemble. The list of ensemble learning algorithms used in this study consists of majority vote, bagging and stacking. A general architecture diagram of the ensembles using multiple SVM models is shown in Fig 4. The aggregation approach is different in each of the ensemble learning algorithms, which will be explained later.

Due to the different algorithms, various training datasets and configurations have been adopted for the purpose of taking advantage of the diversity from the different domains. The configuration of each ensemble can be found in Table 3 and its details are described thereafter. The construction of the ‘others’ training dataset has a few variations:

- others (~200) x 10 means tweets from 10 account owners of the different domains are randomly selected to have a similar size as the samsungsg training dataset, which is of 200 records.
- 10 others means tweets from 10 account owners of the different domains are used individually to combine with the samsungsg dataset to form the training subsets shown in Fig 4.
- others (1978) means all of the tweets from account owners of the different domains are used. The total of 1978 records indicates that some account owners had tweeted less than 200 tweets in the same period (as compared to samsungsg).

**Table 3:** The configuration of various multiple SVM ensembles.

**Serial No.**	**Method**	**Training dataset** ^a^		**Configuration**
1.	SVM with 10 random sampling with majority vote	samsungsg (200)	others (~200) x 10	10 SVM models
2.	SVM with majority vote	samsungsg (200)	10 others	10 SVM models
3.	SVM with bagging	samsungsg (200)	others (1978)	10 SVM models
4.	SVM with stacking	samsungsg (200)	10 others	10 SVM models with Naïve Bayes (kernel) as the tier two classifier

^a^The number in the brackets represents the number of records. The data collection and pre-processing process can be found in S1 Methods.

#### Random Sampling with Majority Vote

**Fig 5:**
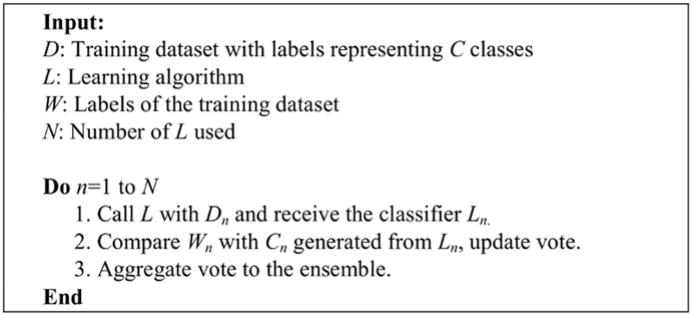
The majority vote algorithm.

One of the simplest solutions to the imbalance data problem is to divide the dataset of the majority class into multiple subsets via random sampling before combining with the minority class to form a balanced training dataset for classification. Individual classifiers are then combined or aggregated by taking a simple majority vote of their decisions (see Fig 5).

Here, random sampling is done on the majority class instead of the minority class (in contrast to the bootstrapping algorithm). The purpose is to divide the majority dataset into subsets of similar sizes as the minority class. Each record in the majority class is randomly selected and placed in a subset until the required size is met. This process of random selection is repeated until all the subsets are created. The records are unique within the same subset but duplicated records can be found across different subsets.

#### Majority Vote

The difference between this method and the random sampling with majority vote method described earlier is the construction of the training dataset. Instead of using random sampling of the entire ‘others’ training dataset, the dataset from each of the account owners is combined with samsungsg’s dataset to form multiple training subsets (see Fig 4). Each of the training subsets is now of similar size and is subsequently used to train an SVM individually. The aggregation approach of this ensemble is through majority vote as described in Fig 5. Similar to the random sampling with majority vote method, the voting is based on labels or classes assigned only. The ensemble then chooses a class that receives the largest total vote and assigns the class as the designated predicted class.

#### Bagging

**Fig 6:**
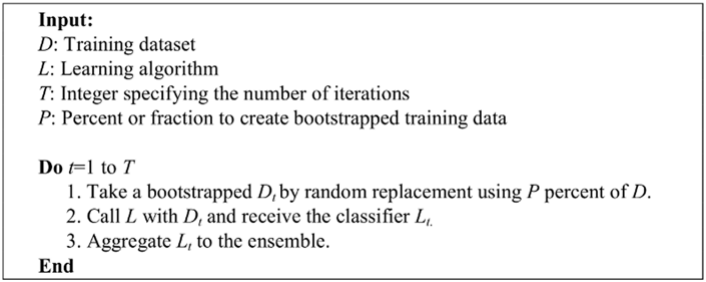
The bagging algorithm.

Bagging is actually an ensemble learning algorithm derived from bootstrapping, hence it is also known as bootstrap aggregating. It is one of the most intuitive and probably the simplest ensemble based algorithm, which generally performs better than other ensemble learning methods [18]. Diversity of classifiers in bagging is achieved by using bootstrapped replicas of the training dataset. The training data subsets are drawn randomly with replacement from the entire training dataset. Each training data subset is used to train a different classifier of the same type. The aggregation approach in bagging is based on majority vote. In other words, the class chosen by most of the classifiers is the ensemble decision. Details of the bagging algorithm are shown in Fig 6.

#### Stacking

**Fig 7:**
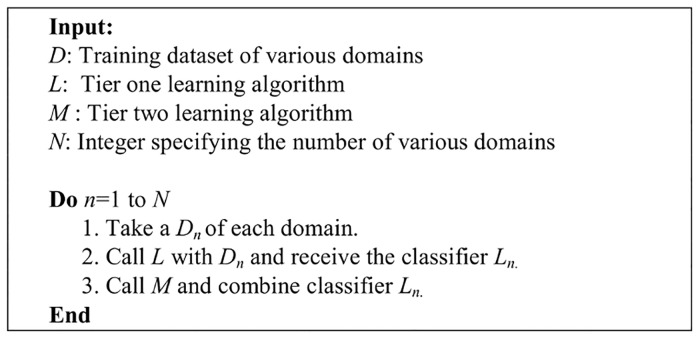
The stacking algorithm.

Stacking or stacked generalisation [19] is a method using an ensemble of two tiers of classifiers to improve the learning process. In [19], an ensemble of classifiers is first trained using bootstrapped samples of the training dataset to create tier one classifiers, which outputs are then used to train the tier two classifier. The intuitive explanation of this method is that it learns the misclassification from tier one classifiers so that the tier two classifier can correct such improper training. In our stacking method, bootstrapping is not used but rather, each training dataset from the 10 account owners is combined with samsungsg to form an individual training subset in order to train the individual SVM (as shown in Fig 4). The aggregation mentioned in Fig 4 for our method is based on the tier two classifier Naïve Bayes (kernel), with a greedy estimation mode of the 10 kernels classifier (built from the 10 training datasets of the 10 account owners and samsungsg) in tier two, to learn from the output of each individual SVM. Naïve Bayes (kernel) has been chosen as the learner because it is a non-parametric estimator that depends on all the outputs of the SVM to reach an estimate, and hence will take into consideration the learning from all the individual SVMs. The underlying idea is similar to the original stacked generalisation method. For example, if a particular classifier had incorrectly learned a certain region of the feature space and consistently misclassified instances from that region, then the tier two classifier may be able to learn from this behaviour. The algorithm used is depicted in Fig 7.

## Experimental Setup

### Data Collection

We have used the Twitter Search API [20] for our data collection. As the API is constantly evolving with different rate limiting settings, our data gathering has been done through a scheduled program that requests a set of data for a given query. Details of the API implementation and query terms (such as showUser() and getUserTimeline()) can be found in S1 Methods under the Supporting Information section. In order to analyse contents of the account owners’ tweets, the most recent 200 tweets by samsungsg and the accounts of the other domains (as specified in the Followers’ Domains Discovery section) between Nov 2, 2012 and Apr 3, 2013 were extracted. A total of 1978 records of the other domains were used in this study. The reason of it being 1978 records instead of 2000 records was due to the fact that some of the account owners had tweeted less than 200 tweets during the specified period. At the time of data collection, there were 3727 samsungsg followers. For each of the followers, the API was used to extract their tweets, giving a total of 187746 records, and 2449 unique users having at least 5 tweets in their past 100 tweets of the same period. We reasoned that those with fewer than 5 tweets were inactive in Twitter, as it implied that these users were tweeting an average of less than one tweet in a month (since the period was of 6 months).

### Performance Metrics

The typical accuracy metric in statistical analysis of binary classification, which takes into consideration the true positive (TP) and true negative (TN), has known issues in terms of reflecting the performance of a classifier [21]. Therefore, we have used the precision, recall, F measure, and G mean as performance metrics when assessing the various SVM ensembles.

The formulas of precision, recall, F measure and G mean are as follows:

precision=TP/(TP+FP) (3)

recallorTruePositiveRate(TPR)=TP/(TP+FN) (4)

TrueNegativeRate(TNR)=TN/(FP+TN) (5)

$$Fmeasure=2\times \frac{precision\times recall}{precision+recall}$$ (6)

$$Gmean=\sqrt{TPR\times TNR}$$ (7)

where TP, TN, FP and FN represent the true positive, true negative, false positive and false negative, respectively.

Both the F measure and G mean have been used as evaluation metrics here instead of the usual average accuracy so that the performance of a classifier with class imbalance can be assessed more accurately. The value of F measure incorporates both precision and recall and hence it is able to measure how good a learning algorithm is for a class. On the other hand, the G mean is based on recalls on both classes and the benefit of selecting this metric is that it can measure how balanced the combination scheme is. If a classifier is highly biased towards one class (such as the majority class), the G mean value is low [7].

In addition, Receiver Operating Characteristic (ROC) analysis and the associated use of the area under the ROC curve (AUC) is another good indicator to assess the overall classification performance.

### Generation of Testing Datasets

In order to assess the performance of the various SVM ensembles, the contents of a total of 300 followers (which were randomly sampled) were annotated manually as either a potential target audience or not a target audience based on the contents shared by the account owner, samsungsg. Even though the original tweet contents from the annotated followers were mostly different, the contents of the testing dataset after the data pre-processing process resulted in a fair amount of duplication. Hence, the testing dataset was further cleaned to remove the duplication so as not to introduce any unnecessary noise to the performance of the classifiers.

A total of 1239 features and 124462 records were used in the study. The features were first extracted from the training dataset through term frequency analysis and word stemming as mentioned previously before using the same features to convert the testing dataset to its feature vectors.

## Results

In this section, results from various experiments are discussed. We first analyse the list of topics/domains identified, followed by showing the inconsistent results from random sampling. Results from 10 fold cross-validation using the various SVM ensembles and their classification of the testing dataset (which is the performance on the previously unseen data) are covered in the latter two sections, respectively.

### Representative Target Topical Words Identified

As shown in Fig 1, tweets from all the followers were pre-processed before they were clustered using Twitter LDA. In order to aid the domain annotation of the human judges, the 60 topic groups were first filtered using the seed words-fuzzy match approach so that topic groups with topical words similar to contents of the target account owner were excluded. From Table 4, we see that the seed words-fuzzy match approach is able to identify 10 topic groups out of the 60 topic groups. This automated approach helps to reduce the annotation efforts, and further manual verification on the topic groups identified (Table 4) indeed shows that the identified topic groups contain topical words related to samsungsg.

**Table 4:** Topic groups identified via the seed words-fuzzy match approach and some of their topical words.

**Topic Model**	**Topic Group Id**	**Topical words**
10	Topic 1	samsung, galaxy, phone, iphone, app, mobile
	Topic 8	singapore, android, ipad, Samsung, sg
20	Topic 9	tv, led, Samsung, contest, giveaway
	Topic 10	galaxy, Samsung, android, tablet, sony, xperia
	Topic 16	samsung, galaxy, android, phone, mobile, iphone, app
30	Topic 0	samsung, galaxy, android, phone, note, iphone, htc
	Topic 2	tv, Samsung, led, video, review, hd
	Topic 12	android, touch, tablet, pc
	Topic 17	galaxy, Samsung, video
	Topic 23	app, google, ipad, android, iphone

The rest of the topic groups with an average score of 0.75 and above can be found in Table 1. As can be seen from that table, localised topical words such as ‘mrt’ (or mass rapid transit, which is the local train service) and ‘changi’ (which represents the name of the international airport in Singapore as well as a popular weekend hangout place called Changi Points) are among those extracted. Some of the topic groups have more than one possible domain, especially for daily musing and hence multiple account owners have been selected to represent the domain.

### Results from Individual SVMs of Random Sampling

**Fig 8:**
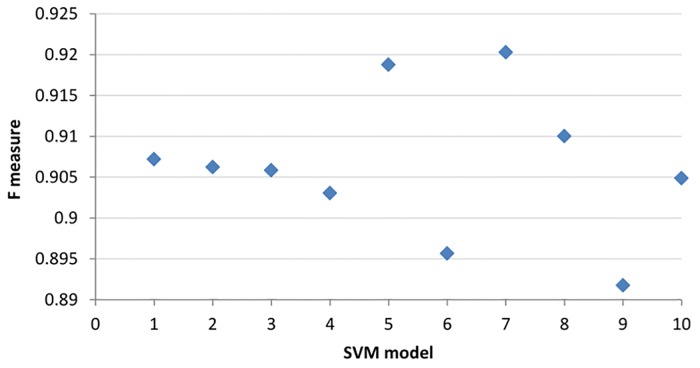
F measures of 10 SVM models generated from random samples. The x-axis represents individual SVM models.

**Fig 9:**
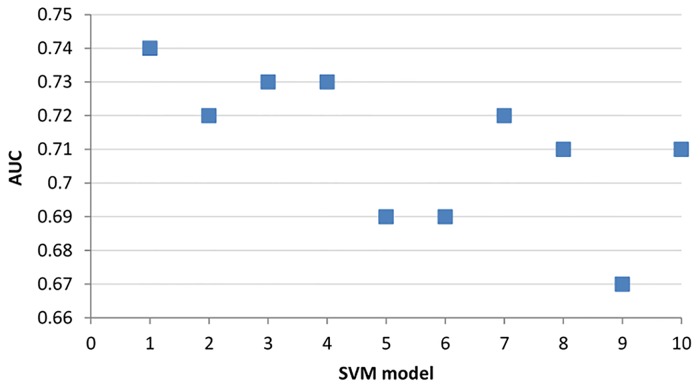
AUC of 10 SVM models generated from random samples. The x-axis represents individual SVM models.

As one of the common approaches for tackling the dataset imbalance issue is under-sampling of the majority class, we used random sampling to continually select a record from a majority class (without duplication) until the size of the minority class was met. While the samples were created mainly to test the performance for the majority vote ensemble method, it is of interest to analyse the results of individual SVMs trained by random samples. 10 fold cross-validation was used to evaluate the performance of each SVM model and the results of F measure and AUC can be found in Figs 8 and 9 respectively. The results clearly show the inconsistency of the model generated by each random sample.

### Training Performance of Various SVM Ensembles

Each of the ensemble methods (including bootstrapping) was evaluated using 10 fold cross-validation. The average performance of 10 runs was recorded and shuffled sampling was used for creating samples for each of the validations. It is observed that the bootstrapping method has produced the best results using performance metrics of recall, precision, F measure and G mean, whereas the ensemble method using random sampling with majority vote has performed the worst. The results of 10 fold cross-validation of various SVM ensembles are listed in Table 5.

**Table 5:** Results of 10 fold cross-validation of various SVM ensembles.

**Method**	**Recall**	**Precision**	**F measure**	**G Mean**
SVM with bootstrapping sampling	1	0.98	0.99	0.99
SVM with 10 random sampling with majority vote	0.31	0.46	0.37	0.54
SVM with majority vote	0.84	0.38	0.52	0.85
SVM with bagging	0.69	0.97	0.80	0.83
SVM with stacking	0.96	0.90	0.93	0.95

### Results of Various SVM Ensembles on the Testing Dataset

While the 10 fold cross-validation results can be a good indicator of the performance of the various classifiers, it is of interest to assess how the various SVM ensembles perform on actual followers’ tweets or previously unseen datasets to assess the ability and the potential of the classifiers to predict or identify a target audience from the list of followers.

**Fig 10:**
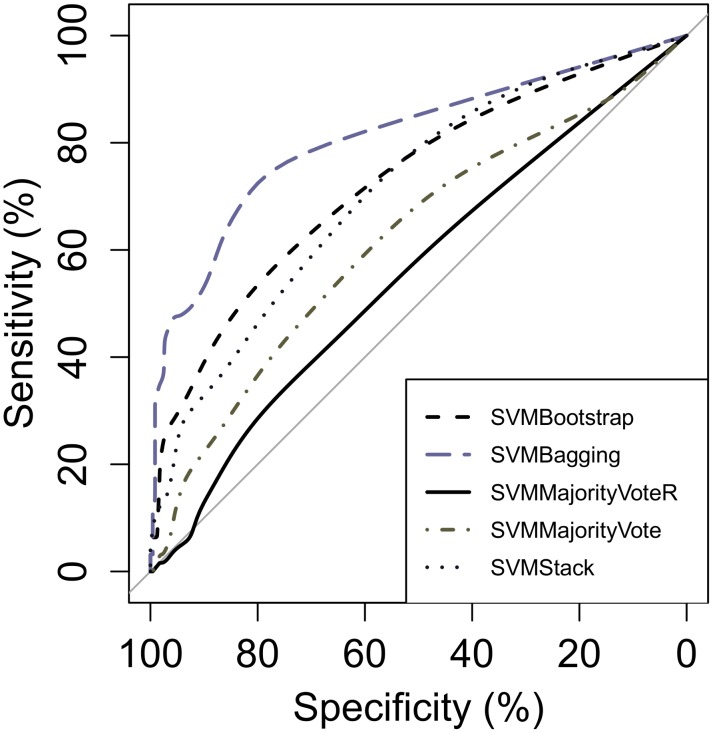
ROC curves of various SVM ensembles on the testing dataset.

The *v* score specified in formula (2) was used to calculate the AUC and plot the ROC curve. In addition, time taken to complete the classification was also recorded. All the experiments were run using the same computer with configurations of a 1.6 GHz processor and an 8 G memory. Each of the experiments was repeated three times to ensure that the time taken and the results are consistent. The results of various SVM ensembles on the testing dataset and the ROC curves can be found in Table 6 and Fig 10, respectively.

**Table 6:** Results of various SVM ensembles on the testing dataset.

**Method**	**AUC**	**Time taken (s)**
SVM with bootstrapping sampling	0.76	1932±61
SVM with 10 random sampling with majority vote	0.62	722±29
SVM with majority vote	0.64	723±16
SVM with bagging	0.89	482±22
SVM with stacking	0.73	629±25

As shown in Table 6, the SVM ensemble with bagging performs the best with an AUC value of 0.89 and time taken of 482 seconds. The bootstrapping method is the next best performer, followed by the stacking method. Both majority vote methods do not perform as well with the random sampling method obtaining only an AUC value of 0.62. This observation is consistent with the F measure and G mean performance metrics shown in Table 5, where the majority vote methods have scored lower as compared to other methods.

## Discussion

It is interesting to observe that, while traditionally a training dataset of the same source is often used for testing purposes, the F measures of SVMs with bootstrapping, bagging and stacking show that tweets from the account owner can be used to identify a target audience. This finding is important, as the proposed approach using both the unsupervised and supervised learning methods eliminates the need to manually annotate the vast amount of tweets from the followers. Using tweets from the owner (which is well categorised within its domain) is more practical if the approach is to be adopted in a real-world application for target audience prediction.

Other approaches for understanding preferences of Twitter users can be found in the relevant literature, e.g., through predicting users’ interests or clustering of users’ demographics, which can be extremely useful for businesses to carry out targeted marketing or personalised services. The majority of these approaches have focused on classifying Twitter users using textual features (e.g., contents of the tweets) [22] or network features (e.g., follower/followee networks) [23]. Michelson and Macskassy [24] presented work in discovering topics of interest by examining the entities in tweets, while Hong et al. [25] modelled a user’s interest and behaviour by focusing on re-tweet actions in Twitter. As most of the Twitter users’ basic demographic information (e.g., gender, age, etc.) is unknown or incomplete (as compared to Facebook), Yang et al. [26] examined the temporal effect of Twitter contents or tweets in classifying users’ interests. Instead of using tweets directly, temporal information is derived from word usage within the streams to boost the accuracy of classification. There are also researchers who have adopted various sociolinguistic features such as emoticon and character repetition, and used the SVM to classify latent attributes such as gender, age, regional origin and political orientation (e.g., see [23]). Ikeda et al. [27] developed some demographic estimation algorithms for profiling Japanese Twitter users based on their tweets and community relationships, where characteristic biases in the demographic segments of users are detected by clustering their followers and followees. None of the related work, however, has attempted to classify a target audience using a combination of unsupervised and supervised methods on a list of followers of an account owner based solely on the contents or tweets shared by the account owner. A direct comparison of performance between our proposed approach and those from the literature is therefore not possible.

While SVM ensembles have been used in common classification problems such as understanding the IRIS dataset [28] or UCI datasets [29], most of the SVM ensemble applications have focused on the bioinformatics domain such as glycosylation site prediction [30] and prediction of microRNA precursors [31]. Previous work using SVM ensembles or combination of an SVM with other types of classifiers in understanding Twitter data can be found in the area of sentiment analysis, in which a bootstrap ensemble framework was proposed to handle both the sentiment multi-class imbalance and data sparsity issues [32] as well as sentiment classification using multiple classifiers and lexicon [33]. Apart from that, Mahmud et al. [34] used tweet contents and tweeting behaviour for home location inference while Shaikh and Padulkar [35] adopted SVM ensembles in topic summarisation. These related previous studies have not focused on target audience classification to identify a social audience leveraging on the data imbalance situation caused by the diversity of followers’ tweets from different domains.

Indeed, one of the easiest ways to “solve” the data imbalance problem is to use a random sample or to generate a random training dataset from the majority class. However, as shown in Figs 8 and 9, this approach is not advisable as the F measure and AUC can be inconsistent among the various SVM models. One of the advantages of using an ensemble method is to minimise the risk of choosing a particularly poor performing classifier from the list of randomly generated models. It is important to highlight that there is no guarantee that the combination of the multiple classifiers will always perform better, but it certainly reduces the overall risk of making a poor selection.

We have observed that the G mean can be a good indicator to assess an ensemble’s performance. While both the majority vote methods (see the Results section) have scored low in the F measure, SVM majority vote that uses the dataset from each of the 10 account owners (instead of random sampling) has a higher G mean. This implies that the method has a more balanced combination and hence is not biased towards any class. As a result, it has performed better in classifying the testing dataset.

Even though the SVM ensemble using bagging has not performed as well in the 10 fold cross-validation using the training dataset compared to bootstrapping or stacking, it is able to generalise well in identifying a target audience from the annotated testing dataset. It has been shown that even though bagging is such a simple ensemble based algorithm, it can have a surprisingly good performance [18]. This is most probably due to two reasons, which are statistically and computationally related. The statistical reason is related to the bootstrapping approach used to address the lack of adequate data to properly represent the data distribution. Table 5 has clearly shown that the SVM ensemble using random sampling does not perform well. The computational reason lies in the majority vote approach in the aggregation of bagging. Since there is no need to select a particular model, it becomes more robust by combining the error reduction and randomness induced by each individual classifier.

In contrast, both bootstrapping and stacking have performed well in the cross-validation of the training dataset but not as well in the classification of the annotated testing dataset. Even though the bootstrapping method adopts a non-parametric approach to statistical inference of over-sampling, it uses a single SVM model to learn the features from the training dataset, and hence may not be able to perform as well on unseen data such as the annotated testing dataset. As for the SVM ensemble built using the stacking algorithm, the tier two classifier is trained to learn the behaviour from multiple tier one classifiers. Hence, it can perform well in cross-validation, as a dataset of similar distribution to the training dataset is used in the evaluation process. It can be argued that contents of the account owner may not be the best choice to construct the training dataset but, considering the elimination of the daunting task of manual annotation of thousands of followers’ tweets and the promising results using SVM ensembles (with the bagging method achieving an AUC value of 0.89), it is definitely worthwhile for any business to adopt this approach of using account owners’ contents for target audience classification.

## Conclusions and Future Work

In this paper, we have demonstrated that by using unsupervised (Twitter LDA) and supervised (SVM ensembles) learning methods, it is possible to automatically classify and identify a target audience from a list of followers of a Twitter account. We have also shown that the account owners’ tweets can be used as the training dataset in an ensemble system for classifying the target audience with minimal annotation efforts.

From the results, we have observed that SVM ensembles, especially using the bagging algorithm, can achieve a high AUC value of 0.89 in target audience classification when classifying a set of unseen, annotated testing data. With the SVM ensembles, the combined result from the various models has the potential to be adapted to any domain as the diversity of the datasets will likely improve the performance instead of being limited by the type of account chosen.

Besides that, our results have also shown that it is essential to adopt a statistical inference approach such as bootstrapping in over-sampling, instead of using random sampling, to construct the training dataset for an SVM ensemble. This conclusion is in agreement with the finding in [7], where it was observed that under-sampling via random sampling has led to lower accuracies. Through this novel way of constructing the training dataset from various account owners for ensemble learning, actionable insights can be uncovered and social media intelligence [36] can be harvested to assist in making better decisions for any company.

Our future work will involve analysing SVM ensembles of other account owners from different domains to verify if the observation is consistent across Twitter. This is important in order to ascertain if such an ensemble method can be generalised to a wide range of domains and hence develop a classifier that is capable of identifying a target audience from the list of followers. Furthermore, it is of interest to analyse if another ensemble method, boosting [7] (such as AdaBoost (Adaptive Boosting) [37]), can perform better in such applications. We would also like to see if the use of biologically inspired Natural Language Processing methods [38], such as the Extreme Learning Machine [39], which has gained increasing popularity recently, can achieve better results in unstructured text analysis. We plan to explore if the combination of different classifiers can improve the classification results. It should be noted that achieving an accuracy of 100% for the application area of targeted marketing is unnecessary as any improvement of mass marketing is going to be beneficial for business companies. Therefore, the results presented here provide an opportunity for businesses to improve the efficacy of their social media programs by identifying potential customers automatically.

## Supporting Information

S1 Methods Supporting methods and processes. (DOC)
