# Supplementary material for: Using Support Vector Machine Ensembles for Target Audience Classification on Twitter
Source: PLoS One. 2015 Apr 13;10(4):e0122855. doi: 10.1371/journal.pone.0122855 (PMC4395415; doi:10.1371/journal.pone.0122855)
Supplement: S1 Methods — (DOC) [file pone.0122855.s001.doc]

**Supporting Information**

**Seed Words Generation**

All the tweets extracted from samsungsg are subjected to a data cleaning and pre-processing process (see the Data Pre-processing section). Each tweet is represented by the identified fragments or words and phrases. This set of data is further processed using term frequency analysis to obtain a list of seed words (which include “samsung”, “galaxy s iii”, “galaxy camera”, etc.). The words in a phrase are joined by ‘_’ so that they can be identified as a single term but the ‘_’ is filtered in the later matching process. In this study, a total of 38 words and phrases have been identified.

**Fuzzy Match**

It is not uncommon for Twitter users to use abbreviations, interjections or different forms of expression to represent a similar term. For example, “galaxy s iii” can be represented by “galaxy s 3”, which is understandable by a human but cannot be captured by direct keyword matching. As such, fuzzy matching based on the seed words derived has been implemented to identify the topic groups with contents similar to the account owner’s.

The comparison here is based on a Dice coefficient string similarity score [1] using the following expression:

$$s=2\times n_{c}/\left(n_{x}+n_{y}\right)$$

where *n_c_* is the number of bigrams found in both strings, *n_x_* is the number of bigrams in string x and *n_y_* is the number of bigrams in string y. For example, to calculate the similarity between “process” and “proceed”:

x = process bigrams for x = {pr ro oc ce es ss}

y = proceed bigrams for y = {pr ro oc ce ee ed}

Both x and y have 6 bigrams each, of which 4 of them are the same. Hence, the Dice coefficient string similarity score is 2*4/(6+6) = 0.667. A similarity score of above 0.7 is considered as a match in identifying the topic groups that are relevant to the target domain. Topic groups with 3 or more topical words with a similarity score higher than 0.7 are excluded.

**Data Pre-processing**

Tweets are known to be noisy and often mixed with linguistic variations. It is hence very important to clean up the tweet content prior to any content extraction:

- Non-English tweets were removed using the Language Detection Library for Java [2];
- URLs, any Twitter’s username found in the content (which is in the format of @username) and hashtags (with the # symbol) were removed;
- Each tweet was pre-processed to lower case.

As tweets are usually informal and short (up to 140 characters), abbreviation and misspelling are often part of the content and hence the readily available Named Entity Recognition package may not be able to extract relevant entities properly. Due to this, we have derived an approach called Entities Identification, which uses Part-of-Speech (POS) [3] tags to differentiate the type of words. All the single nouns are identified as possible entities. If the tag of the first fragment detected is ‘N’ (noun) or ‘J’ (adjective) and the consecutive word(s) is of the ‘N’ type, these words will be extracted as phrases. This approach is then complemented by another process using the comprehensive stop words list used by search engines (<http://www.webconfs.com/stop-words.php>) in addition to a list of English’s common words (preposition, conjunction, determiners) as well as Twitter’s common words (such as “rt”, “retweet”, etc.) to identify any possible entity. In short, the original tweet is sliced into various fragments by using POS tags, stop words, common words and punctuations as separators or delimiters. For example, if the content is “Samsung is holding a galaxy contest!”, two fragments will be generated for the content as follows: (samsung) | (galaxy contest).

**Twitter API Implementation**

The Twitter Search API is implemented through Twitter4J API [4] in our study. A Twitter object, twitter, is first created before requesting for authorisation from Twitter. Once an access token has been granted, two different programs are used to extract the relevant data. The first program is developed to extract information about a Twitter user, which includes the user id and followers count. Specifically, the code User user = twitter.showUser(username) is adopted and a set of accessor methods such as getId() and getFollowersCount() are implemented. The second program is used to retrieve the date and content of tweets based on the Twitter username. In addition to the user object extracted earlier, a list of status containing dates (using getCreatedAt()) and contents (using getText()) of tweets is obtained through List<Status> statuses = twitter.getUserTimeline(username ,paging), where paging is set to 200 for the top 200 tweets.

**References**

1. Kondrak, G., Marcu, D., Knight, K.: Cognates can improve statistical translation models. Proceedings of the 2003 Conference of the North American Chapter of the Association for Computational Linguistics on Human Language Technology. pp. 46-48. Association for Computational Linguistics (2003).

2. Nakatani, S.: language-detection - Language Detection Library for Java - Google Project Hosting, http://code.google.com/p/language-detection/.

3. Toutanova, K., Manning, C.D.: Enriching the knowledge sources used in a maximum entropy part-of-speech tagger. Proceedings of the 2000 Joint SIGDAT Conference on Empirical Methods in Natural Language Processing and Very Large Corpora. pp. 63-70. Association for Computational Linguistics (2000).

4. Yamamoto, Y.: Twitter4J, http://twitter4j.org/.
